# CALORIC RESTRICTION VERSUS TIME-RESTRICTED EATING: THE HEALTH, AGING, AND LATER-LIFE OUTCOMES PILOT

**DOI:** 10.1093/geroni/igae098.1052

**Published:** 2024-12-31

**Authors:** Denise Houston, Barbara Nicklas, Michael Miller, Jason Fanning, Fang-Chi Hsu, James DeLany, Jamy Ard, Stephen Kritchevsky

**Affiliations:** Wake Forest University School of Medicine, Winston Salem, North Carolina, United States; Wake Forest University School of Medicine, Winston-Salem, North Carolina, United States; Wake Forest University School of Medicine, Winston-Salem, North Carolina, United States; Wake Forest University, Winston-Salem, North Carolina, United States; Wake Forest University School of Medicine, Winston-Salem, North Carolina, United States; AdventHealth, Orlando, Florida, United States; Wake Forest University School of Medicine, Winston-Salem, North Carolina, United States; Wake Forest University School of Medicine, Wake Forest University School of Medicine, North Carolina, United States

## Abstract

In animal models, dietary interventions including caloric restriction (CR) and time-restricted eating (TRE) extend both lifespan and healthspan. However, in humans, the long-term benefits of these diets on lifespan and healthspan, and whether benefits outweigh harms, are unknown. The goal of the Health, Aging and Later-Life Outcomes Pilot (HALLO-P) is to inform the design of a definitive, full-scale trial to evaluate the long-term effects of CR and TRE in older adults. HALLO-P randomized 90 older (≥ 60 yrs) adults with obesity, or overweight and at least one comorbidity, to assess the feasibility and acceptability of three different 9-month interventions: 1) 20% CR delivered in-person; 2) 20% CR delivered remotely; and 3) 8-hour TRE with ad libitum caloric intake. Recruitment is complete and final intervention and close-out will be completed in August, 2024. To date, retention is 89% and intervention session attendance is 84%. Those assigned to CR are expected to weigh daily – to date, weights were obtained on 90% percent of days – and expected to maintain their weight within a zone of adherence – 65% of CR participants achieved this >75% of the time. TRE participants are expected to eat within a 7.5-8.5 hour window – TRE participants reported eating within an 8.5-hour window 77% of the time. Final study data will include: 1) the degree of sustained CR (using doubly-labeled water); 2) the sustainability of TRE (number of participants eating within the 8-hr window); and 3) changes in body weight and composition, and metabolic and functional health.

